# CRISPR/Cas9-mediated targeted mutagenesis in grape

**DOI:** 10.1371/journal.pone.0177966

**Published:** 2017-05-18

**Authors:** Ikuko Nakajima, Yusuke Ban, Akifumi Azuma, Noriyuki Onoue, Takaya Moriguchi, Toshiya Yamamoto, Seiichi Toki, Masaki Endo

**Affiliations:** 1 Institute of Fruit Tree and Tea Science, National Agriculture and Food Research Organization, Fujimoto, Tsukuba, Ibaraki, Japan; 2 Institute of Agrobiological Sciences, National Agriculture and Food Research Organization, Kannondai, Tsukuba, Ibaraki, Japan; 3 Graduate School of Nanobioscience, Yokohama City University, Seto, Kanazawa-ku, Yokohama, Kanagawa, Japan; 4 Kihara Institute for Biological Research, Yokohama City University, Maioka-cho, Yokohama, Kanagawa, Japan; Universidade de Lisboa Instituto Superior de Agronomia, PORTUGAL

## Abstract

RNA-guided genome editing using the CRISPR/Cas9 CRISPR (clustered regularly interspaced short palindromic repeats)/Cas9 (CRISPR-associated protein 9) system has been applied successfully in several plant species. However, to date, there are few reports on the use of any of the current genome editing approaches in grape—an important fruit crop with a large market not only for table grapes but also for wine. Here, we report successful targeted mutagenesis in grape (*Vitis vinifera* L., cv. Neo Muscat) using the CRISPR/Cas9 system. When a Cas9 expression construct was transformed to embryonic calli along with a synthetic sgRNA expression construct targeting the *Vitis vinifera* phytoene desaturase (VvPDS) gene, regenerated plants with albino leaves were obtained. DNA sequencing confirmed that the VvPDS gene was mutated at the target site in regenerated grape plants. Interestingly, the ratio of mutated cells was higher in lower, older, leaves compared to that in newly appearing upper leaves. This result might suggest either that the proportion of targeted mutagenized cells is higher in older leaves due to the repeated induction of DNA double strand breaks (DSBs), or that the efficiency of precise DSBs repair in cells of old grape leaves is decreased.

## Introduction

Functional analysis of any gene of interest in plant genomes has traditionally relied heavily on the use of transfer DNA (T-DNA) and transposon insertional mutagenesis, or chemical- and irradiation-induced mutagenesis, to generate mutants. However, saturation mutagenesis is difficult in non-model plants due to a lack of genome information, large genome size and/or low transformation efficiency. Recent progress in the use of sequence-specific nucleases (SSNs) has opened new opportunities for reverse genetics in plants. SSNs, which include zinc finger nucleases (ZFNs), transcription activator-like effector nucleases (TALENs), and the clustered regularly interspaced short palindromic repeat (CRISPR)/CRISPR-associated 9 endonuclease (Cas9) system, have been developed to induce DNA double-strand breaks (DBSs) at specific genome sites. DSBs have a high propensity to induce site-directed mutations through error-prone genome repair via non-homologous end joining (NHEJ). Once transgenic cells expressing SSN constructs are obtained, various mutations will be induced at the specific target locus in independent cells, meaning that the limitations of low transformation efficiency can be overcome by the high performance of SSNs.

Of the SSNs now available, the CRISPR/Cas9 system has been utilized successfully for mutagenesis in a variety of organisms, including plants such as Arabidopsis, sorghum, rice, tomato, maize, wheat, potato, poplar, orange, liverwort, petunia, and cucumber (for review, see Bortesi and Fischer [1]). In addition, very recently, successful CRISPR/Cas9 mediated targeted mutagenesis in grape was reported by Ren et al. [2]. Unlike ZFNs or TALENs, the CRISPR/Cas9 system does not require any protein engineering steps, making it much more straightforward to test multiple single guide RNAs (sgRNAs) for each target gene. Furthermore, only 20 nt in the sgRNA sequence need to be changed to confer a different target specificity, which means that complicated cloning is also unnecessary. This allows the inexpensive assembly of large sgRNA libraries so that the CRISPR/Cas9 system can be used for high-throughput functional genomics applications, bringing genome editing within the budget of any molecular biology laboratory.

Grape is one of the most important deciduous fruit crops worldwide. Total world grape production in 2012 was estimated at 67 million tons, which ranks 9th in agricultural production terms, not counting livestock [3]. In 2007, 65% of total grape production, estimated at 271 million hectoliters, was used in wine production [4]. Because of its high economic value, many traits involved in aroma, disease and stress response, color of fruit skin, and size of fruit, are of interest in grape. The whole-genome sequence of *Vitis vinifera* was released in 2007 [5], and the ability to produce transgenic grape is a further valuable research tool for studying and understanding the genetics and function of the genes and processes involved in disease and pest resistance and plant development, as well as primary and secondary metabolism [6]. There are several reports of transformation and regeneration in grape (*Vitis vinifera* L., *V*. *labruscana* Bailey) [7–15]. Although a few successful transformations using biolistic methods have been reported [7,8], *Agrobacterium*-mediated transformation of embryogenic tissue is by far the most popular method for generating transgenic grape [9–15]. Somatic embryogenic tissues can be induced from anthers [16,17], unfertilized ovules [17–19], filaments [20] and leaves [17,21,22]. Transgenic approaches offer a means of inserting new characters into the genome of traditional cultivars. However, cultivated grape maintains high levels of heterozygosity, and back crossing to eliminate somaclonal mutation and/or cross breeding to introduce valuable features is not the preferred route for grape breeding. From this point of view, pin-point modification of endogenous genes using the CRISPR/Cas9 genome editing system is an effective method for both functional analysis and breeding of new varieties. Some reports on the application of CRISPR/Cas9 in grape have now been published. Ren et al. [2] used suspension cells of the wine grape ‘Chardonnay’ as a material for *Agrobacterium*-mediated transformation of Cas9 and sgRNA expression constructs, targeting the endogenous l-idonate dehydrogenase (IdnDH) gene. In a computational study, Wan et al. [23] identified specific target sites for CRISPR/Cas9 in the widely cultivated grape species *V*. *vinifera*, and developed a database for editing grape genomes. Most recently, DNA-free genome editing of grape protoplasts was reported [24]. The authors of the latter report delivered purified CRISPR/Cas9 ribonucleoproteins (RNPs) to protoplasts of the grape cultivar ‘Chardonnay’ and achieved an estimated mutation frequency in protoplasts of 0.1% (as assessed by targeted deep sequencing). Experimental conditions were substantially different in all the grape CRISPR/Cas9 studies cited above, thus precluding any direct comparison of mutation efficiency in different studies. The purpose of our present study was to demonstrate the universality of CRISPR/Cas9-mediated targeted mutagenesis in grape. We used embryogenic calli of the table grape ‘Neo Muscat’ for *Agrobacterium*-mediated transformation of Cas9 and sgRNA expression constructs targeting the phytoene desaturase (PDS) gene. Because PDS deficiency leads to a visible bleaching phenotype, the PDS gene is selected as a target gene for CRPSPR/Cas9-mediated genome editing in many plant studies. *V*. *vinifera* is a diploid plant, and bi-allelic mutations in the PDS gene change the leaf color from green to white. Taking advantage of this visible phenotype, we were able to show a correlation between leaf age and mutation ratio. Our study complements other grape CRISPR/Cas9 studies, and will be informative for grape molecular breeding.

## Materials and methods

### *In vitro* cleavage assay

All steps were performed according to the manufacturer’s instructions provided in the Guide-it Complete sgRNA Screening System (Takara Clontech, Japan) with a few modifications, as follows: as a cleavage reaction, we mixed 100ng of cleavage template, 20 ng of synthesized sgRNA, 250 ng of Cas9 nuclease in 10 μl of 1x Cas9 reaction buffer with 1x BSA. Primers used for amplifying cleavage templates and expected band sizes are as follows:

Template for PDS-t1: VvPDS-F1 5’-TCGGAAAGTTGTTGGGATTGTTGGAAGAGAAGA-3’, VvPDS-R1 5’-AGCATTACAGATAATTACTTAGCAACACGGAATGT-3’, Non-digested, 1372bp; digested, 610bp and 762bp.
Template for PD-t2: VvPDS-F2 5’-TCCATTGGTCTTATCATGTCCTTGAGGCATGGCT-3’, VvPDS-R2 5’-TGATTGGTTGGGTTCTAGATGAGGGCAGGTAAGT-3’, Non-digested, 1300bp; digested, 534bp and 766bp.
Template for PDS-t3 and PDS-t4: VvPDS-F3 5’-AGCATATGTGAAGATGCAGGCCTTGAATTTGGGGA-3’, VvPDS-R3 5’-TCTTGCTTGCATATCAGGAGGAAGTACCAGCCCAT-3’, non-digested 1328bp; digested at PDS3, 335bp and 993bp; digested at PDS4, 916bp and 412bp.

### Vector construction

Oligonucleotide pairs for the target sequences were annealed, and the resulting fragments cloned into the *Bbs*I site of the sgRNA cloning vector pUC19_AtU6oligo, in which the attL1::AtU6-26::gRNA::PolyT::attL2 fragment from pEn-Chimera [25] lies between the two I-*Sce* I sites of the vector. To complete the all-in-one binary vector harboring sgRNA, Cas9 and an NPTII expression construct, we used pZK_OsU3gYSA_Cas9, which contains a Cas9 expression cassette prepared from pDe-Cas9 [25], an NPTII expression cassette, and sgRNA expression construct OsU3::gYSA. The sgRNA expression cassette, AtU6-26::gRNA::PolyT, prepared in pUC19_AtU6oligo, was excised at the I-*Sce* I sites and replaced by OsU3::gYSA in pZK_OsU3gYSA_Cas9, completing pZK_gPDS-t2_Cas9 and pZK_gPDS-t3_Cas9.

### Plant material and transformation of grape with Cas9 and sgRNA expression plasmids

**Fig 1:**
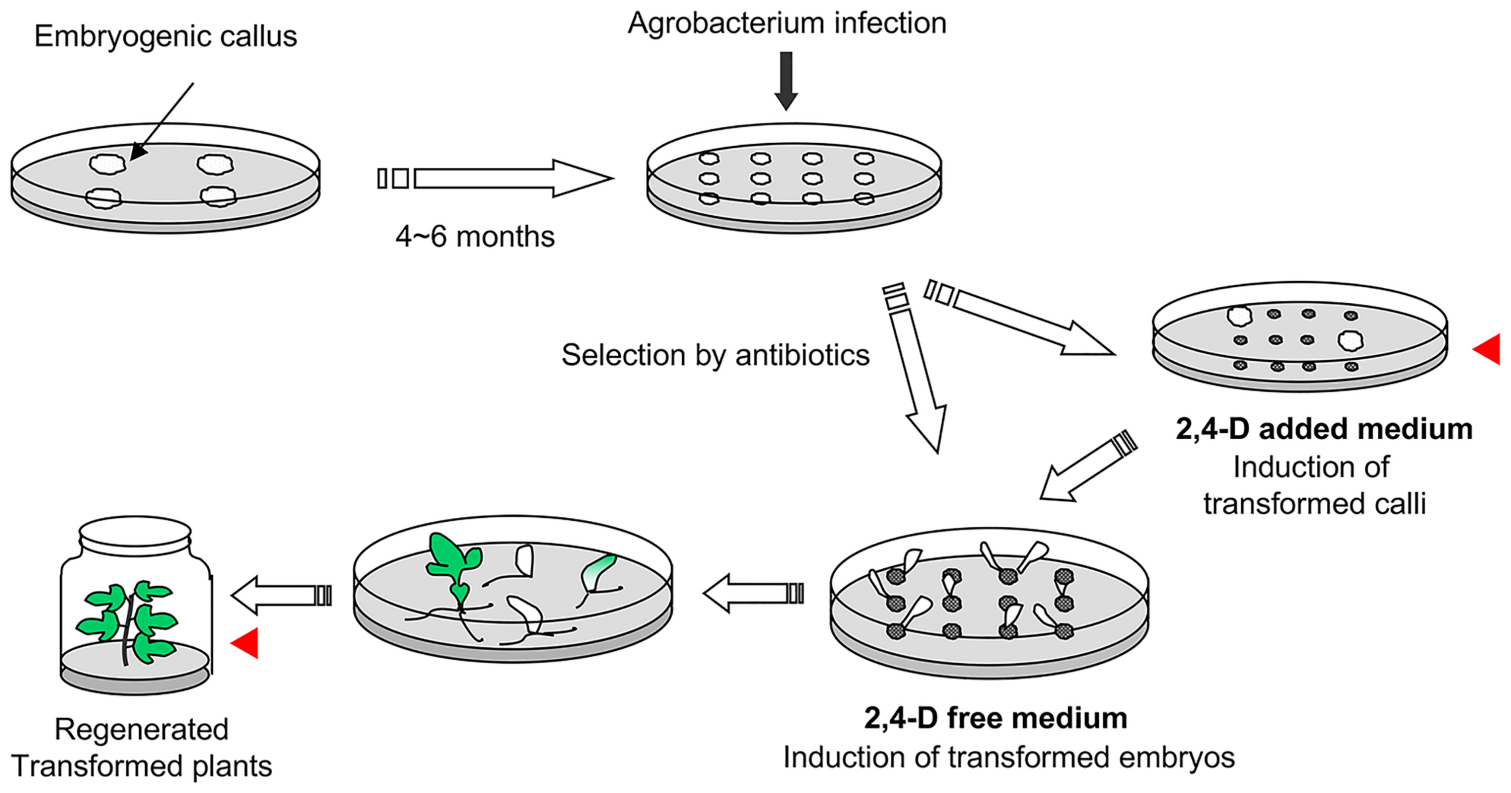
Schematic representation of transformation and regeneration process. After *Agrobacterium*-mediated transformation, calli were transferred to callus induction medium or regeneration medium. Red arrowheads indicate the point of mutation detection. Materials used for mutation analysis are shown by red arrowhead.

A schematic representation of how the transgenic plants were obtained is shown in Fig 1, with full details given in S1 Fig. Immature inflorescences (about 2–3 weeks before anthesis) of the table grape cultivar, ‘Neo Muscat’ (*V*. *vinifera* L.) collected from a vineyard were sterilized with sodium hypochlorite (1% available chlorine) containing a few drops of Tween 20 for 20 minutes, then rinsed twice with sterile distilled water. Filaments were cultured as described previously [20]. Briefly, filaments were excised from buds under aseptic conditions. About 10 filaments were cultured in a 100 ml conical flask containing 25 ml of half-strength MS basal liquid medium [26] with 1 μM 2,4-dichlorophenoxyacetic acid (2,4-D) and 1 μM *N*-(1, 2, 3-thiadiazol-5-yl)-*N'*-phenylurea (thidiazuron) (TDZ), adjusted to pH 5.8 before autoclaving. The flasks were agitated continuously in a rotary shaker (60 rpm). All explants were cultured at 26°C in the dark. After 1 month of culture, filaments were transferred to 1/2 MS solid medium containing 1 μM 2,4-D, 1 μM TDZ and 0.85% agar, and cultured for 2 months. Induced embryogenic cultures were transferred to fresh NN medium [27] containing 1 μM 2,4-D and 1.5% agar once a month; 4–6 months later, proliferated embryonic calli were used for *Agrobacterium*-mediated transformation. Plasmids were introduced into *Agrobacterium tumefaciens* strain LBA4404 by electroporation according to the supplier’s instructions. *Agrobacterium* was incubated in YEB medium containing spectinomycin and rifampicin at 28°C on an orbital shaker at 140 rpm overnight. Culture media were centrifuged at 6000 rpm for 5 min. The pellet was resuspended in 1/2 MS medium (5% maltose instead of sucrose, pH 5.8) containing 100 μM acetosyringone, and adjusted to 1 x 10^8^ cfu. Embryogenic calli were immersed in bacterial suspension in a Petri dish, vacuumed for 5 minutes for air exclusion between calli, and thereafter infected for 15 minutes. After removing excess bacterial suspension with sterile filter paper, calli were transferred to co-cultivation medium (1/2 MS medium, 5% maltose instead of sucrose, 1 μM 2,4-D and 100 μM acetosyringone, pH 5.8) and incubated for 5 days at 26°C in the dark. After co-cultivation, calli were rinsed with 1/2 MS medium (5% maltose instead of sucrose, pH 5.8) containing 400 μg/ml cefotaxime. To induce transformed embryogenic calli, infected calli were transferred to selection medium (callus induction medium with 1/2 MS medium (5% maltose instead of sucrose, 1.5% agar) containing 1 μM 2,4-D, 50 μg/ml kanamycin and 200 μg/ml cefotaxime, and incubated in the dark for 3–4 months. To induce transformed embryos, calli were transferred to regeneration medium with 1/2MS medium (5% maltose instead of sucrose, 1.5% agar), 25 μg/ml kanamycin and 200 μg/ml cefotaxime, and incubated in the dark for 3–4 months. Calli were transferred to fresh medium once a month. Induced somatic embryos were then transferred to germination medium [1/2MS medium (3% sucrose, 2% agar) containing 5 μM zeatin], and incubated in conditions of 16h light / 8h dark to induce true leaves.

### Detection of CRISPR/Cas9-induced genome editing

Genomic DNA was extracted from calli and regenerated plants using a DNeasy Plant Mini Kit (Qiagen, Germany) according to the supplier’s instructions. PCR analysis was conducted to detect transgenes in kanamycin-resistant calli.

The primer pair AtU6p-F and sgRNAs-R, corresponding to AtU6pro to sgRNA scaffold regions, was used for PCR amplification to confirm integration of the AtU6sgRNA into calli and regenerated plants. Other primer pairs for the Cas9 (Cas-F and Cas-R) and NPTII (NPTII-F and NPTII-R) region were also used. PCR amplification was performed in a total volume of 20 μl containing 10 ng of genomic DNA, 2 μl of 10 μM primers, and 10 μl of GoTaq DNA polymerase (Promega, USA). Thermal cycler conditions were as follows; 95°C for 2 min; followed by 40 cycles of 30 sec at 94°C, 30 sec at 55°C, and 1.5 min at 72°C; then 5 min at 72°C. Amplifications were performed with the GeneAmp PCR system 9700 (Applied Biosystems, USA).

Primer pairs (PDS-t2F and PDS-t2R) and (PDS-t3F and PDS-t3R) were used to amplify the target region of PDS-t2 and PDS-t3, respectively. PCR amplification was performed in a total volume of 20 μl containing 10 ng of genomic DNA, 0.5 μl of 10 μM primers, 2 μl of 10×buffer, 2 μl of 2 mM dNTP, 2 μl 25mM MgCl_2_ and 0.3 μl of KOD plus neo (TOYOBO, Japan). Thermal cycler conditions were as follows; 94°C for 2 min; followed by 40 cycles of 10 sec at 98°C, 30 sec at 55°C, and 30 sec at 68°C. To detect mutation, PCR products from callus DNA were digested with restriction enzymes *Ssp* I (PDS-t2) and *Taq* I (PDS-t3), because CRISPR/Cas9-mediated mutation disrupts the recognition sequences of these restriction enzymes. Digested PCR products were analyzed by agarose gel electrophoresis. Accurate mutation frequency and mutation pattern were identified as follows: non-digested PCR products were ligated into pCR-Blunt II-TOPO (ThermoFisher, USA) using a Zero Blunt TOPO PCR cloning kit (Invitrogen, USA) and transformed to *Escherichia coli* DH5α (TaKaRa, Japan). Plasmids were isolated using a Wizard Plus SV minipreps DNA Purification System (Promega, USA), and subjected to sequence analysis using a BigDye X-Terminator 3.1 cycle sequencing kit (Applied Biosystems, USA) and an ABI3130x sequencer (ThermoFisher, USA). The primer pair M13F / M13R was used for sequence analysis, while the primer pair chr13-F and chr13-R, chr17-F and chr17-R, chr19-F and chr19-R were used for amplification of first, second and third off-target candidates site of PDS2.

### Western blot analysis

Leaves of regenerated plants were ground with equal amounts of protein extraction buffer, and the soluble fraction was separated by centrifugation; 20 μl of protein extract was fractionated by SDS-PAGE on a 5–20% Tris-glycine SDS gradient pre-cast polyacrylamide gel (ATTO, JAPAN), and subjected to immuno-blotting with Cas9 antibody (Active motif, USA) using immunoreaction enhancer solution Can Get Signal (TOYOBO, Japan). After rinsing with 1xTBST, blots were hybridized with second-antibody (stabilized goat anti-mouse IgG HRP-conjugated; Thermo Fisher Scientific, USA), and signals were detected using Super Signal West Dura Maximum Sensitivity Substrate (PIERCE, USA) and the ChemiDoc Touch Imaging system (Bio-Rad, USA).

## Results

### Selection of target sequences on the *VvPDS* gene

**Fig 2:**
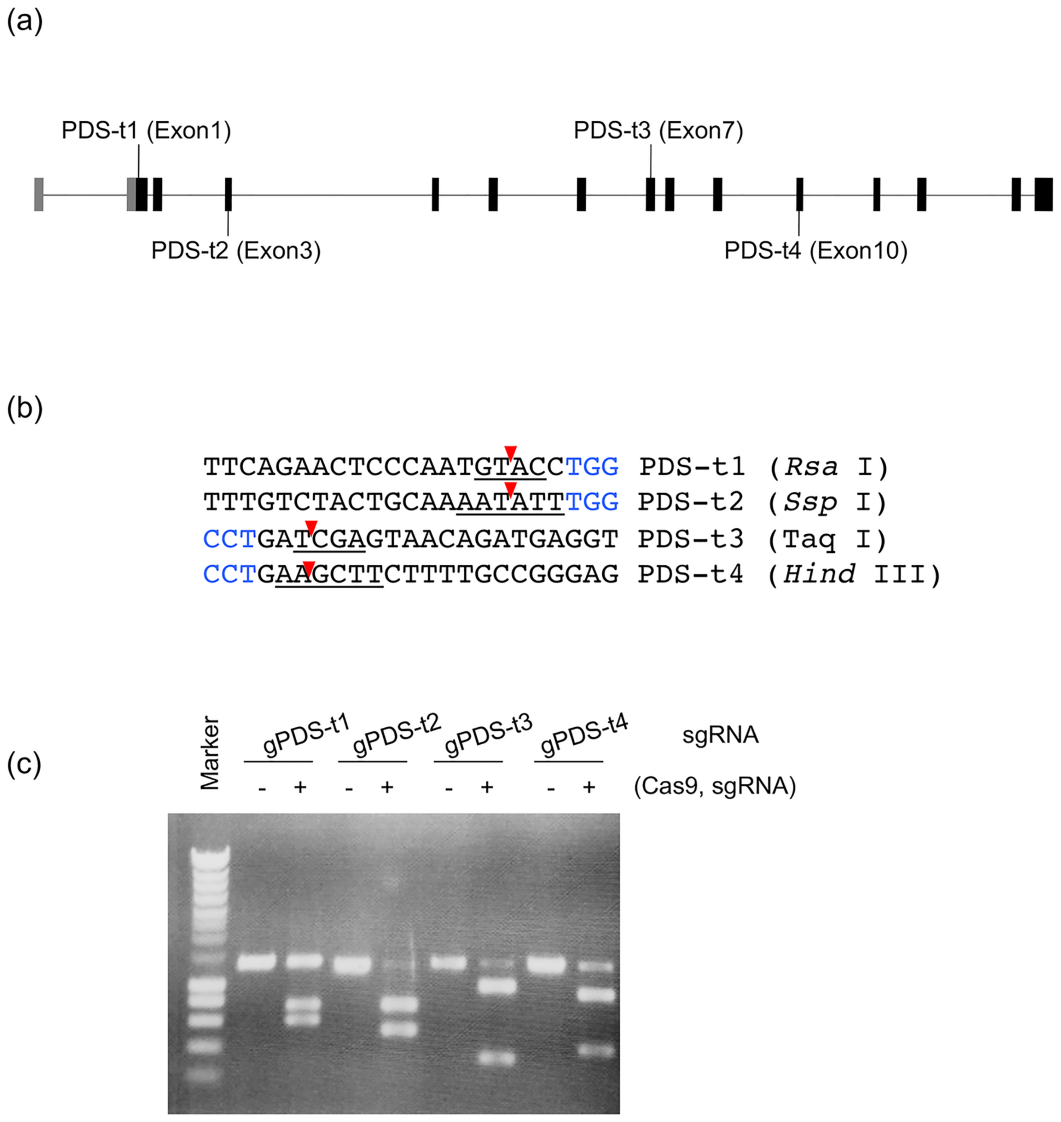
Selection of target sites in the *VvPDS* gene. (a) Locations of target sites of CRISPR/Cas9-mediated target mutagenesis in the ‘Neo Muscat’ (*V*. *vinifera* L.) PDS gene. (b) Sequences of target sites. The PAM sequence (NGG) is shown in blue, and the 20bp target sequence is shown in black. The red arrowhead indicates the expected cleavage site. The recognition sequences of the restriction enzymes used for CAPS analysis are underlined. (c) *In vitro* cleavage assay of four target sites.

To test whether CRISPR/Cas9-mediated targeted mutagenesis system functions in grape, four different sites on *VvPDS* exons were targeted for cleavage (Fig 2a and 2b). These target sequences were selected by first identifying the NGG protospacer adjacent motif (PAM) sequence required for *Streptococcus pyogenes* Cas9, and then capturing the 20 nucleotides immediately upstream of the PAM sequence for use as the spacer in the sgRNA. In a previous study, we found that mutation efficiency varied at different target sequences in rice [28]. An *in vitro* cleavage assay was conducted to exclude inferior sgRNAs. The designed sgRNA molecules can be generated by *in vitro* transcription. When the sgRNAs, Cas9, and PCR fragments containing the target sequence were mixed and incubated, we observed cleavage of the target DNA in all four sgRNA constructs (Fig 2c). The cleavage efficiency of gPDS1 seemed lower than with the other sgRNAs, while that of gPDS2 and gPDS3 seemed to be slightly higher than that of gPDS4. Thus, we decided to use gPDS2 and gPDS3 for targeted mutagenesis in grape.

### CRISPR/Cas9-mediated targeted mutagenesis in grape callus

**Fig 3:**
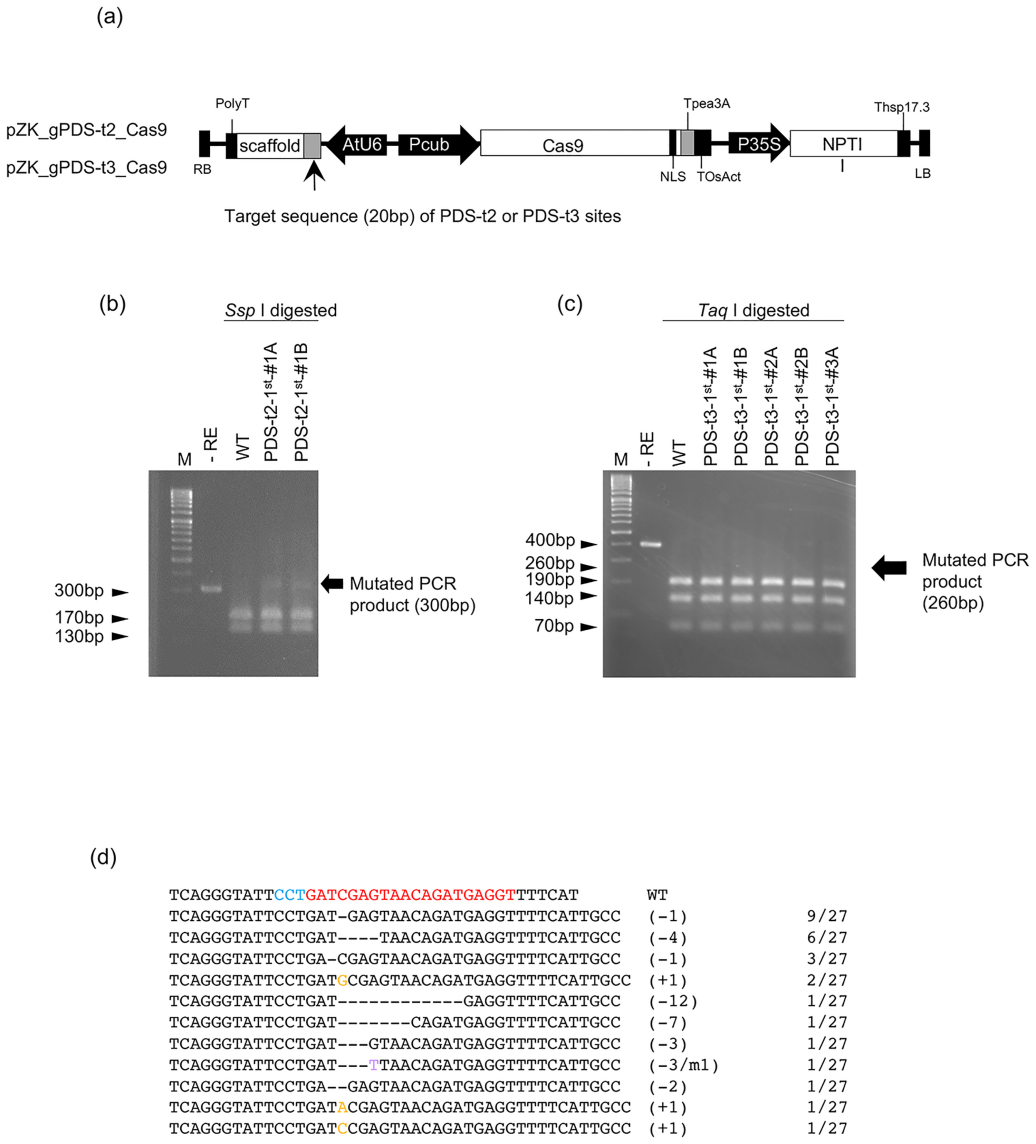
Detection of mutations in the PDS gene in Cas9-, sgRNA-transformed calli. (a) Expression constructs for Cas9 and sgRNA. (b, c) CAPS analysis of the *PDS* locus. DNA extracted from independent pZK_gPDS-t2_Cas9 (b) and pZK_gPDS-t3_Cas9 (c) transformed calli was subjected to PCR and subsequent restriction enzyme digestion. M, Marker;–RE, PCR product without restriction enzyme reaction; WT, restriction enzyme digested PCR product of wild-type grape DNA. In addition to the target site of PDS-t3, another *Taq* I site exists on the amplicon of the PDS-t3 target sequence. Appearance of a 260-bp band after *Taq* I digestion indicates CRISPR/Cas9-mediated mutation on PDS-t3 target site. (d) Representative sequences of mutant alleles identified in calli of PDS-t3-1^st^-#3A. The wild-type sequence is shown at the top with the PAM sequence highlighted in cyan, and the target sequence in red. Dashes, deleted bases. The net change in length is noted to the right of each sequence (+, insertion;—deletion). The number of clones representing each mutant allele is shown in the column on the right.

Binary vectors harboring Cas9, and sgRNAs targeting PDS-t2 or PDS-t3 with the selection marker gene *NPTII* (pZK_gPDS-t2_Cas9 and pZK_gPDS-t3_Cas9, Fig 3a) were transformed to grape embryogenic calli induced from filaments of pre-anthesis flowers. Inoculated calli were then transferred to callus propagation medium containing 2,4-dichlorophenoxyacetic acid (2,4-D), kanamycin and cefotaxime (callus induction method), or regeneration medium containing only kanamycin and cefotaxime (embryo induction method) for selecting transgenic cells and to kill *Agrobacterium* (Fig 1). In the callus induction method, kanamycin-resistant calli were proliferated for 3–4 months after *Agrobacterium* infection. Thereafter, these calli were transferred to regeneration medium for 5 months, and regenerated embryos were transferred to germinating medium to regenerate plants. In the embryo induction method, inoculated calli were transferred directly to regeneration medium containing kanamycin, and transgenic embryos were induced directly. These regenerated embryos were then transferred to germinating medium to regenerate plants. The time spent in a dedifferentiated state in the callus induction and embryo induction methods was 11 months and 8–10 months, respectively.

In experiment 1 (Exp. 1—callus induction method), one (PDS-t2-1^st^-#1) and three (PDS-t3-1^st^-#1–3) kanamycin-resistant calli were obtained from 72 infected calli in both cases with pZK_gPDS-t2_Cas9 and pZK_gPDS-t3_Cas9, respectively (Table 1). The presence of *NPTII*, sgRNA expression constructs targeting PDS-t2 (or 3) and Cas9 genes was confirmed by PCR using DNA samples extracted from small pieces of calli (data not shown). At 5 months after transformation, transgenic calli were analyzed to detect targeted mutagenesis. DNA was extracted from two isolated regions of calli, named PDS-t2-1^st^-#1A, B, PDS-t3-1^st^-#1A, B and PDS-t3-1^st^-#2A, B, because, even in the clonally propagated Cas9 /sgRNA transformed callus, targeted mutagenesis occurred stochastically. In the case of PDS-t3-1^st^-#3A, DNA was isolated from a single region only because proliferation of callus was not so vigorous. The expected cleavage sites of PDS-t2 and PDS-t 3 lie within recognition sequences for the restriction enzymes *Ssp* I and *Taq* I, respectively. Restriction sites will be disrupted if CRISPR/Cas9 has cleaved the target sequence successfully; thus, cleaved amplified polymeric sequences (CAPS) were used to detect mutations. Indeed, *Ssp* I non-digested PCR products were detected in two DNA samples derived from PDS#2–1, (Fig 3b, PDS2-1^st^-#1A and PDS2-1^st^-#1B). In the case of PDS-t3, another *Taq* I site existed on the PCR product, and CRISPR/Cas9-mediated mutation results in a 260-bp band after *Taq* I digestion. We found this 260-bp fragment indicating targeted mutation in one transgenic callus, PDS-t3-1^st^-#3A (Fig 3c), and cloned this fragment into pCR-Blunt II-TOPO (Invitrogen) for sequence analysis. When 27 independent clones were analyzed, 11 different mutations were detected (Fig 3d). Considering the fact that 260-bp *Taq* I-digested PCR products were not dominant, the mutation ratio in callus itself was relatively low. However, the occurrence of mutation in calli means that non-chimeric plans with the desired mutation can be obtained when regenerated plants are induced from mutated cells.

**Table 1:** Primer sequences.

Primer name	Primer sequences
PDS-t2F	ACCATAAAATGATTATGTAATGCAAA
PDS-t2R	TGTCTTATAATAACCAATAAGGGGAGA
PDS-t3F	GGCGCAAGCAATGTTGTAG
PDS-t3R	CCAAAATCAATTTCAATGGTCA
NPTII-F	GGCTATTCGGCTATGACTGG
NPTII-R	CATGTGTCACGACGAGATCC
Cas-F	TCGAGAAGATGGATGGAACC
Cas-R	CTGAAACCTGAGCCTTCTGG
AtU6p-F	TGTTTATCAGCTTACATTTTCTTGAACCGTAGCT
sgRNAs-R	TAATGCCAACTTTGTACAAGAAAGCTGGGTCTAGA
chr13-F	TGTTGATTATAATTTTTTCATATTT
chr13-R	AAAACCAAATGTAACTGTTACTTC
chr17-F chr17-R chr19-F chr19-R	CCACTAAAATGTAGAAATGGGG TTCTACATCCAAGCAATTGC ATGATGAATTCATGTAAGCTGC ACCCTCTTTTCCCGACTTTC
M13F	GTAAAACGACGGCCAG
M13R	CAGGAAACAGCTATGA

### Detection of CRISPR/Cas9-mediated mutations in regenerated grape plants

**Fig 4:**
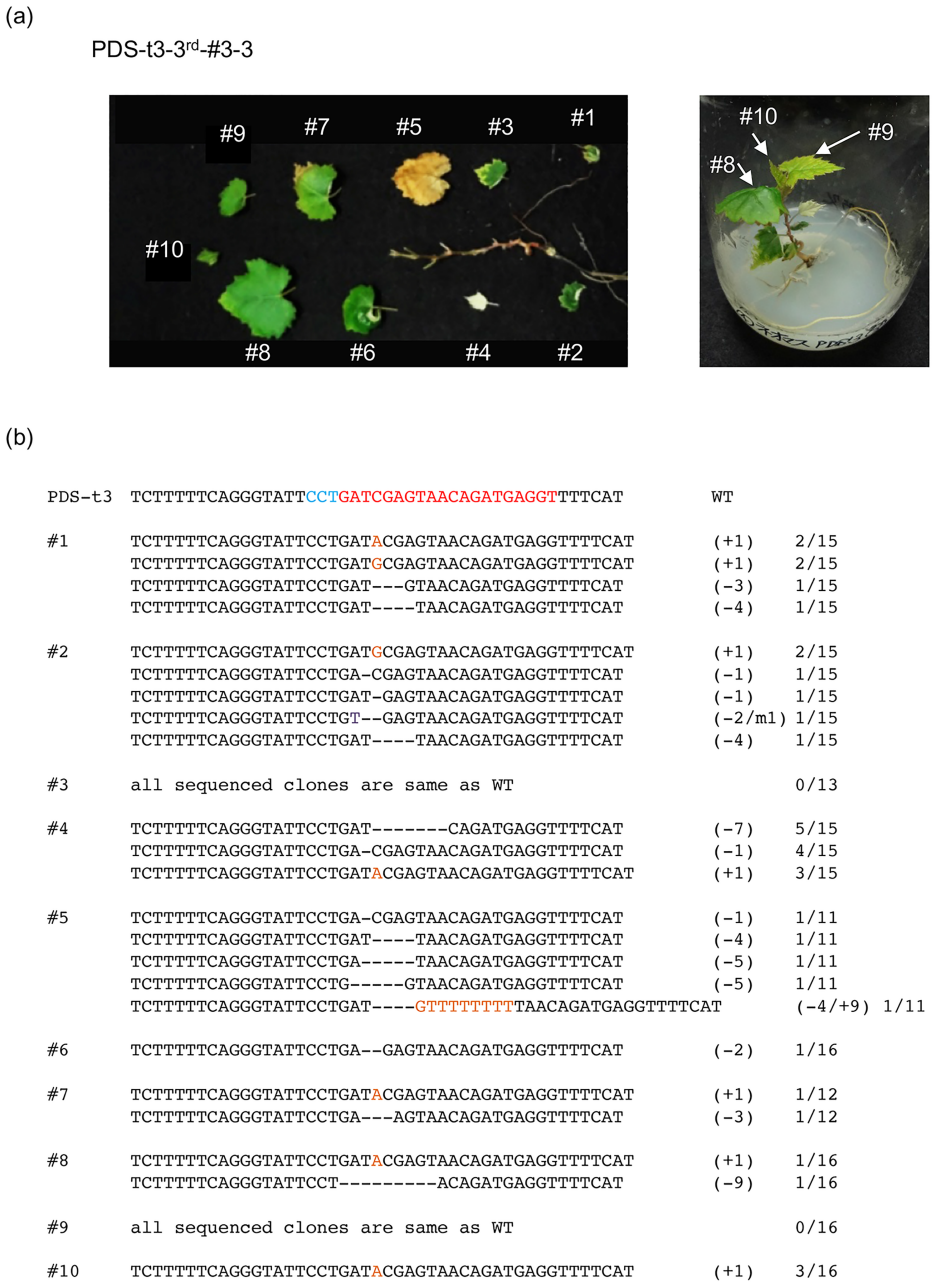
Detection of mutations at the PDS-t3 target locus in regenerated plants. (a) Chlorophyll-deficient variegated plant due to mutation in the PDS-t3 target locus. (b) Representative sequences of the PDS-t3 target locus in leaves. The wild type sequence is shown at the top with the PAM sequence highlighted in cyan, and the target sequence in red. Dashes, deleted bases. The net change in length is noted to the right of each sequence (+, insertion;—deletion). The number of clones representing each mutant allele is shown in the column on the right.

**Fig 5:**
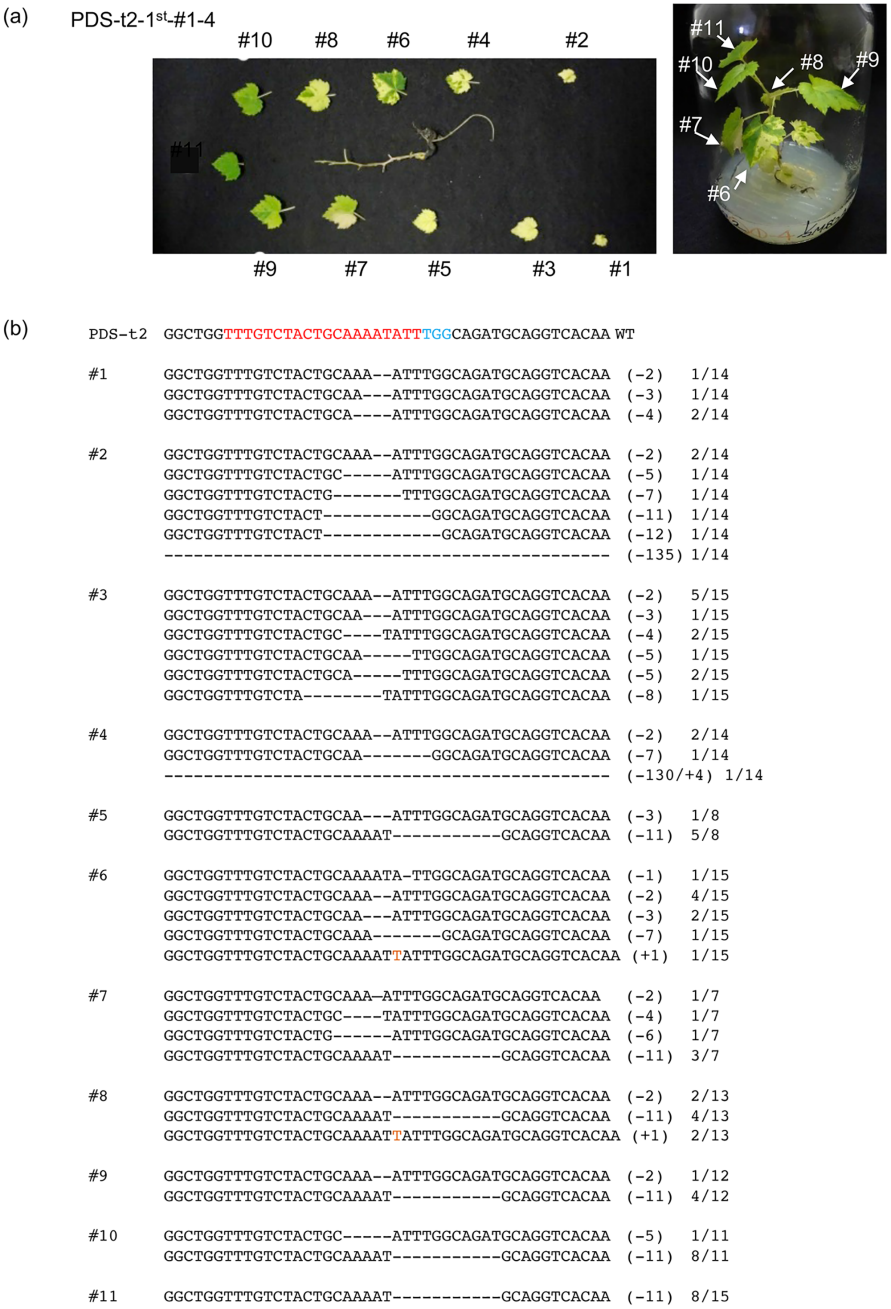
Detection of mutations in PDS-t2 target locus in regenerated plant. (a) Chlorophyll-deficient variegated plant due to mutation in the PDS-t2 target locus. (b) Representative sequences of PDS-t2 target locus in leaves. The wild type sequence is shown at the top, with the PAM sequence highlighted in cyan, and the target sequence in red. Dashes, deleted bases. The net change in length is noted to the right of each sequence (+, insertion;—deletion). The number of clones representing each mutant allele is shown in the column on the right.

From Exp. 1 (callus induction method), 22 and 37 regenerated plants were obtained for PDS-t2 and PDS-t3, respectively (Table 2). From Exps. 2 and 3 (embryo induction method), 60 and 18 regenerated plants were obtained for PDS-t2 and PDS-t3, respectively (Table 3). No pale green or white leaves appeared in wild-type regenerated plants without transformation (S2 Fig). On the other hand, parts of leaves turned pale green or white in some of these transgenic plants, suggesting disruption of the PDS gene. We isolated DNA from independent leaves and analyzed the mutations. In PDS-t3-3rd#3–3 obtained from Exp. 3, only a few leaves had pale green or white parts (Fig 4a), although when the PCR products of each leaf were cloned and sequenced, mutated sequence was detected in 8 out of 10 leaves (Fig 4b). The exact proportion of mutated cells in each leaf was calculated from the ratio of cloned PCR products with mutation. The proportions of mutated DNA in PDS-t3-3rd#3–3 leaf nos. 1–10 were as follows; #1, 40% (6/15); #2, 40% (6/15); #3, 0% (0/13); #4, 80% (12/15); #5, 45% (5/11); #6, 6.3% (1/16); #7, 17% (2/12); #8, 12.5% (2/16); #9, 0% (0/16); and #10, 19% (3/16). Similar to the mutations detected in calli (Fig 3d), small deletions at the target site were frequently observed, but single nucleotide insertions were also often detected. Mutation rates in lower leaves, which appear at an earlier stage of development in regenerated plants, tend to be higher than in upper leaves. In regenerated plant PDS-t2-1st-#1–4 obtained from Exp. 1, chlorophyll deficiency could be seen in large numbers of leaves (Fig 5a). The proportions of mutated DNA in leaf nos. 1–11 were as follows; #1, 29% (4/14); #2, 50% (7/14); #3, 80% (12/15); #4, 29% (4/14); #5, 75% (6/8); #6, 60% (9/15); #7, 86% (6/7); #8, 62% (8/13); #9, 42% (5/12); #10, 82% (9/11); and #11, 53% (8/15). In the two latter plants, multiple mutated sequences were detected. In newly emerged upper leaves, chlorophyll deficiency was not so clear, with ratios and the variation in mutations observed being more limited compared to those of lower, older, leaves. This tendency was observed in other regenerated plants; an example of another plant is shown in S3 Fig. These results indicate that CRISPR/Cas9-mediated targeted mutagenesis occurred stochastically in developing leaves, and that accumulation of bi-allelic mutated cells altered the leaf color in older leaves.

**Table 2:** Emergence rate of mutated plants via callus induction method.

Target	Agrobacterium infected calli	Km-resistant calli	Regenerated plants	Regenerated plants with chlorophyll deficiency	Ratio of plants with chlorophyll deficiency (%)
PDS-t2 (Exp.1)	72	1	22	16	72.2
PDS-t3 (Exp.1)	72	3	37	1	2.7

**Table 3:** Emergence rate of mutated plants via embryo induction method.

Target	Agrobacterium infected calli	Regenerated plants	Regenerated plants with chlorophyll deficiency	Ratio of plants with chlorophyll deficiency (%)
PDS-t2 (Exp.2)	21	29	8	27.6
PDS-t2 (Exp.3)	58	31	1	3.2
PDS-t3 (Exp.3)	72	18	2	11.1

### Confirmation of Cas9 protein expression in transgenic grape leaves

**Fig 6:**
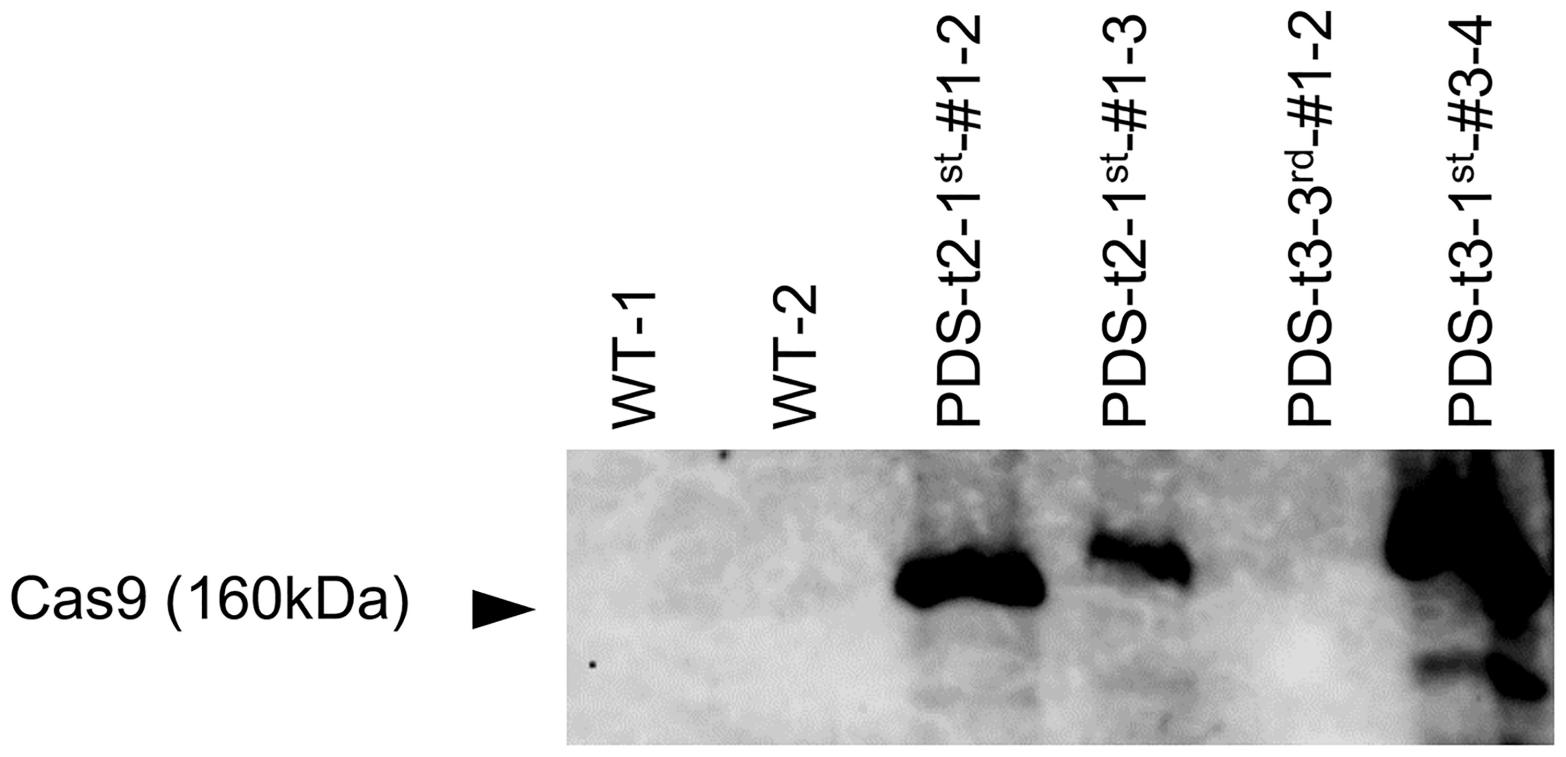
Detection of Cas9 expression in regenerated plants. Immunological detection of Cas9. PDS-t2-1^st^-#1–2 and PDS-t2-1^st^-#1–3 are regenerated plants obtained from same transgenic line.

The mutation rate and degree of bleaching were different in each regenerated plant even though the same CRISPR/Cas9 construct was transformed. To analyze the relationship between Cas9 expression level and the bleaching phenotype, western blot analysis was conducted using a Cas9 antibody. Crude proteins were extracted from two non-transgenic plants, and two transgenic plants of CRISPR/Cas9 vector targeting PDS2 and PDS3, respectively. Among these four transgenic plants, only PDS-t3-3^rd^-#1–2 lacked a noticeable bleaching phenotype (S4 Fig). Cas9 immunodetection revealed that the expression level of Cas9 was lower in PDS-t3-3^rd^-#1–2 than in the other transgenic plants (Fig 6), indicating that Cas9 expression level affects mutation frequency.

### Analysis of potential off-target mutations

Potential off-target sequences were identified using the CRISPR-P program ([29] http://cbi.hzau.edu.cn/cgi-bin/CRISPR). In the case of PDS-t2, the candidate sequence with the highest potential for off-target mutation is 5’-TTTGTCTACTGCAACATATT TGG-3’, which has a 1-nt mismatch. Because high expression levels of Cas9 and sgRNA induce both on-target and off-target mutation [28], we used leaf no. 5 of PDS-t2-1^st^-#1–4 (Fig 5a and 5b), which showed the highest on-target mutation frequency (8/13, 61.5%) for off-target mutation analysis. When the PCR products of this highest potential off-target candidate site amplified by primers, chr13-F and chr13-R (Table 1) were cloned and sequenced, no mutated clones were found (0/15 sequenced clones, data not shown). We also analyzed the possibility of mutations in the second (5’- TGTCTCTGCTGCAAAATATT TGG-3’) and third (5’-TTTGTTTACTGGAAAATATT AAG-3’) potential off-target sites, but no-mutated clones were found in 19 and 26 sequenced clones, respectively.

## Discussion

The present study demonstrated successful CRISPR/Cas9-mediated targeted genome mutagenesis in grape. Mutation frequencies and analyses of mutation patterns suggested that the stably transformed Cas9/sgRNA expression constructs used in this study can induce targeted mutations efficiently in grape leaves. The frequency of mutated cells was higher in old lower leaves relative to newly appearing upper leaves (Figs 4 and 5, S3 Fig). We reported the correlation between on-target mutation efficiency and Cas9 and gRNA expression levels in a previous study [28]. However, expression levels of ubiquitin4-2 in old parsley leaves is known to be lower than that in new leaves [30], so there is only a low possibility of high expression of Cas9 driven by the parsley ubiquitin4-2 promoter in old grape leaves. One hypothesis for explaining this difference is that DNA cleavage and precise DNA repair at the breakage sites are repeated in old leaves, and thus the chance of inducing *de novo* mutations increases due to aging. In other words, mutations might be induced preferentially in old leaves since the chances of DSB repair error could be increased due to prolonged repeated DSBs or decreased efficiency of precise DSB repair in cells of old leaves. In this respect, Boyko et al. [31] revealed that DSB repair mechanisms are developmentally regulated in *Arabidopsis*, whereby the proportion of breaks repaired via homologous recombination decreases substantially as the plant matures.

In this study, we followed two strategies to regenerate grape plants after transformation of the Cas9/sgRNA expression vector. The first was a callus induction method in which transformed embryogenic calli were kept growing on callus induction medium for 3–4 months before transfer to regeneration medium. The other strategy was an embryo induction method in which somatic embryos are induced just after transformation. In rice, which is often used as a model plant for targeted mutagenesis studies, mutations were induced efficiently in calli, and T0 plants regenerated from mutated cells are in a non-chimeric state for the desired mutation. On the other hand, in grape callus, the desired mutations were detected at only a low rate 4 months after transformation, and no non-chimeric (non-mosaic) mutated grape plants were obtained with this induction method. Because the desired mutations were induced mainly after regeneration, and because prolonged tissue culture has disadvantages such as increased somaclonal mutations and reduced regeneration ability, the embryo induction method seems to be better in our case. In the other successful grape genome editing study reported to date [2], suspension cells induced from anthers of ‘Chardonnay’ were used for *Agrobacterium*-mediated transformation of the Cas9/sgRNA expression construct. In this case, the transgenic cell mass was a chimeric mix of mutated and non-mutated cells and bi-allelic mutated plants were also not obtained.

Grape is a seed-propagated plant, but the heterogeneity of the genome in each cultivar will be changed by sexual reproduction. For this reason, dissolution of the chimeric state of mutated and non-mutated cells and obtaining homozygous plants of desired mutations by genetic segregation is not the preferred route for grapes in many cases. Regeneration of plantlets via embryogenic callus induced from mutated cells in leaves is one choice for obtaining non-chimeric mutated grape plants. Matsuta and Hirabayashi [21] reported successful regeneration of intact plants from embryogenic callus derived from mature leaves in grape. Another alternative is the induction of mutations in asexual embryos and/or lateral buds by using embryo-specific promoters for Cas9 and sgRNA expression. In fact, meristem-specific promoters, such as CLAVATA3, APETARA1 and INCURVATA2 have been used to express engineered nuclease, TALENs and Cas9 to increase the heritability of mutations [32–34]. sgRNAs are typically expressed from Pol III promoters such as U6 and U3. However, sgRNA driven by an RNA polymerase II promoter can induce mutations, and expression of both Cas9 and sgRNAs from a single Pol II promoter has also led to induced mutations [35].

For the functional analysis of target genes, any Cas9 and sgRNA expression constructs remaining in grape plants will not usually cause problems. However, for the breeding of new grape cultivars, transgene-free genome editing methods are preferable. There are several ways to create transgene-free mutated plants, including transient expression of the nuclease components using agro-infiltration or viral vectors, or the delivery of components directly as functional sgRNA and Cas9 protein. Woo et al. [36] succeeded in targeted mutagenesis by direct introduction of pre-assembled sgRNA:Cas9 complexes into plant protoplasts of lettuce, *Arabidopsis*, tobacco, and rice. This system is particularly advantageous because it does not fall under the regulatory scrutiny faced by transgenic crops, as the resulting plants are devoid of transgenes encoding CRISPR/Cas9 machinery. Improvements in the CRISPR/Cas9 system itself, as well as new expression strategies, will thus accelerate molecular breeding in grape.

## Supporting information

S1 Fig Details of transformation and regeneration process. Materials used for mutation analysis are shown by red arrowheads.
(PDF)

S2 Fig Regenerated plants of non-transformed ‘Neo Muscat’. Bleached areas are less wide in leaves.
(PDF)

S3 Fig Detection of mutations in the PDS-t2 target locus in regenerated plant. (a) Chlorophyll-deficient variegated plant due to mutation in the PDS-t2 target locus. (b) Representative sequences of the PDS-t2 target locus in leaves. The wild type sequence is shown at the top, with the PAM sequence highlighted in cyan, and the target sequence in red. Dashes, deleted bases. The net change in length is noted to the right of each sequence (+, insertion;—deletion). The number of clones representing each mutant allele is shown in the column on the right. #5 leaf was divided in two parts: green (#5–1) and pale green (#5–2).
(PDF)

S4 Fig Appearance of regenerated plants used for western blot analysis. Bleaching or pale green cells were dominant in three regenerated plans (PDS-t2-1^st^-#1–2, 3 and PDS-t3-1^st^-#3–4). On the other hand, leaf color of PDS-t3-3^rd^-#1–2 is almost the same as that of wild-type, ‘Neo Muscat’ (S2 Fig).
(PDF)
