# Supplementary material for: CRISPR/Cas9-mediated targeted mutagenesis in grape
Source: PLoS One. 2017 May 18;12(5):e0177966. doi: 10.1371/journal.pone.0177966 (PMC5436839; doi:10.1371/journal.pone.0177966)
Supplement: S1 Fig — Materials used for mutation analysis are shown by red arrowheads. (PDF) [file pone.0177966.s001.pdf]

# S1 Fig

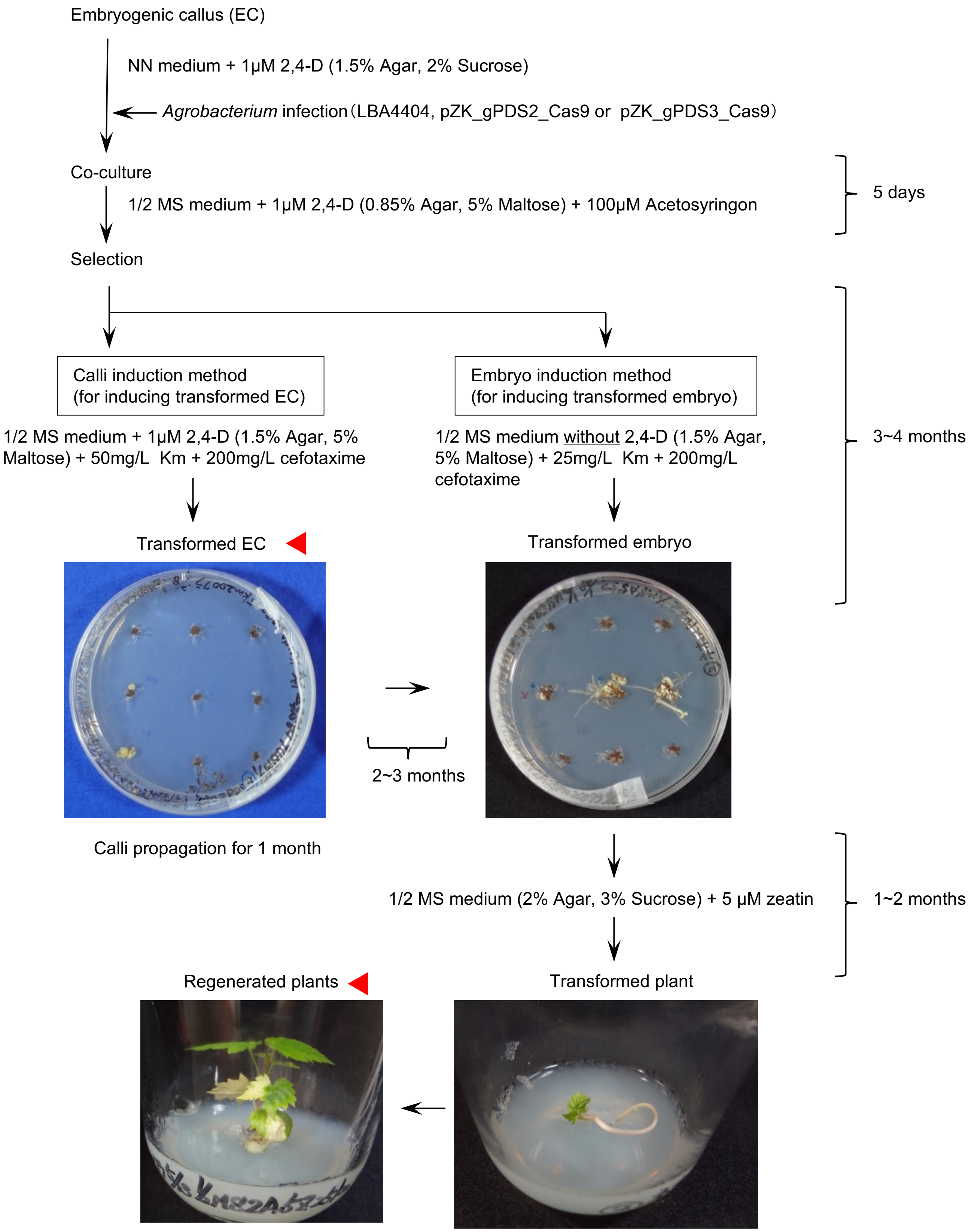


**S1 Fig. Details of transformation and regeneration process.**
Materials used for mutation analysis are shown by red arrowheads.
