# Supplementary material for: CRISPR/Cas9-mediated targeted mutagenesis in grape
Source: PLoS One. 2017 May 18;12(5):e0177966. doi: 10.1371/journal.pone.0177966 (PMC5436839; doi:10.1371/journal.pone.0177966)
Supplement: S2 Fig — Bleached areas are less wide in leaves. (PDF) [file pone.0177966.s002.pdf]

S2 Fig

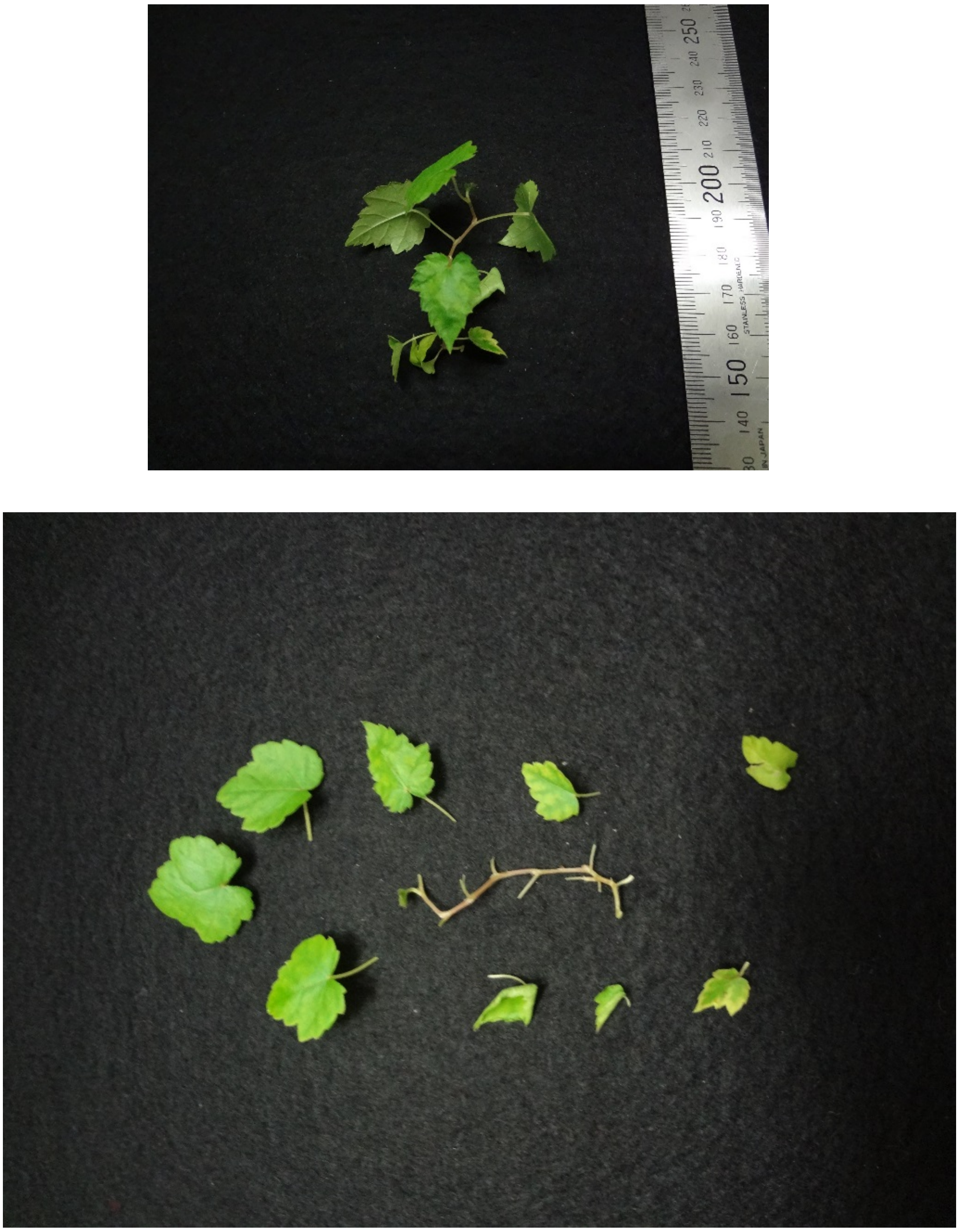

**S2 Fig. Regenerated plants of non-transformed 'Neo Muscat'.**
Bleached areas are less wide in leaves.
