# Supplementary material for: CRISPR/Cas9-mediated targeted mutagenesis in grape
Source: PLoS One. 2017 May 18;12(5):e0177966. doi: 10.1371/journal.pone.0177966 (PMC5436839; doi:10.1371/journal.pone.0177966)
Supplement: S3 Fig — (a) Chlorophyll-deficient variegated plant due to mutation in the PDS-t2 target locus. (b) Representative sequences of the PDS-t2 target locus in leaves. The wild type sequence is shown at the top, with the PAM sequence highlighted in cyan, and the target sequence in red. Dashes, deleted bases. The net change in length is noted to the right of each sequence (+, insertion;—deletion). The number of clones representing each mutant allele is shown in the column on the right. #5 leaf was divided in two parts: green (#5–1) and pale green (#5–2). (PDF) [file pone.0177966.s003.pdf]

S3 Fig

(a) PDS-t2-2nd-#1-5

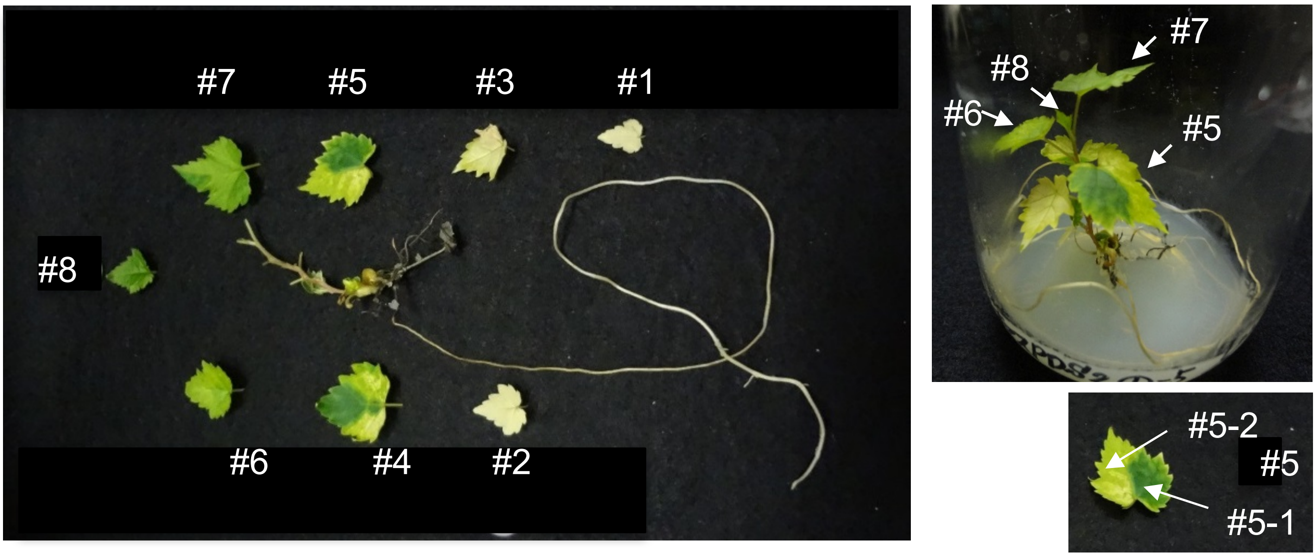

(b)

```
PDS#2   GGCTGGTTTGTCTACTGCAAAATATTTGGCAGATGCAGGTCACAA WT

#1      GGCTGGTTTGTCTACTGCAA-----TTGGCAGATGCAGGTCACAA (-5)    1/12

#2      GGCTGGTTTGTCTACTGCAAA--ATTTGGCAGATGCAGGTCACAA (-2)    1/14
        GGCTGGTTTGTCTACTGCAA---ATTTGGCAGATGCAGGTCACAA (-3)    1/14
        GGCTGGTTTGTCTACTGC----TATTTGGCAGATGCAGGTCACAA (-4)    1/14
        GGCTGGTTTGTCTACTGCAAAATTATTTGGCAGATGCAGGTCACAA        (+1)    1/14

#3      GGCTGGTTTGTCTACTGCAAA--ATTTGGCAGATGCAGGTCACAA (-2)    2/12
        GGCTGGTTTGTCTACTG------ATTTGGCAGATGCAGGTCACAA (-6)    1/12
        GGCTGGTTTGTCTACTG-------TTTGGCAGATGCAGGTCACAA (-7)    1/12
        ----------------------------------------------        (-131) 1/12

#4      GGCTGGTTTGTCTACTGCAAA--ATTTGGCAGATGCAGGTCACAA (-2)    3/15
        GGCTGGTTTGTCTACTGC-----ATTTGGCAGATGCAGGTCACAA (-5)    1/15

#5-1    GGCTGGTTTGTCTACTGCAAA--ATTTGGCAGATGCAGGTCACAA (-2)    1/14
#5-2    GGCTGGTTTGTCTACTGCAAA--ATTTGGCAGATGCAGGTCACAA (-2)    4/11
        GGCTGGTTTGTCTACTGCA----ATTTGGCAGATGCAGGTCACAA (-4)    1/11
        GGCTGGTTTGTCTACTGC----TATTTGGCAGATGCAGGTCACAA (-4)    1/11
        GGCTGGTTTGTCTACTGCAAAATTATTTGGCAGATGCAGGTCACAA(+1)    1/11

#6      all sequenced clones are same as WT
#7      all sequenced clones are same as WT
#8      all sequenced clones are same as WT
```

S3 Fig. Detection of mutations in the PDS-t2 target locus in regenerated plant.
(a) Chlorophyll-deficient variegated plant due to mutation in the PDS-t2 target locus. (b) Representative sequences of the PDS-t2 target locus in leaves. The wild type sequence is shown at the top, with the PAM sequence highlighted in cyan, and the target sequence in red. Dashes, deleted bases. The net change in length is noted to the right of each sequence (+, insertion; - deletion). The number of clones representing each mutant allele is shown in the column on the right. #5 leaf was divided in two parts: green (#5-1) and pale green (#5-2).
