# Supplementary material for: CRISPR/Cas9-mediated targeted mutagenesis in grape
Source: PLoS One. 2017 May 18;12(5):e0177966. doi: 10.1371/journal.pone.0177966 (PMC5436839; doi:10.1371/journal.pone.0177966)
Supplement: S4 Fig — Bleaching or pale green cells were dominant in three regenerated plans (PDS-t2-1st-#1–2, 3 and PDS-t3-1st-#3–4). On the other hand, leaf color of PDS-t3-3rd-#1–2 is almost the same as that of wild-type, ‘Neo Muscat’ (S2 Fig). (PDF) [file pone.0177966.s004.pdf]

S4 Fig

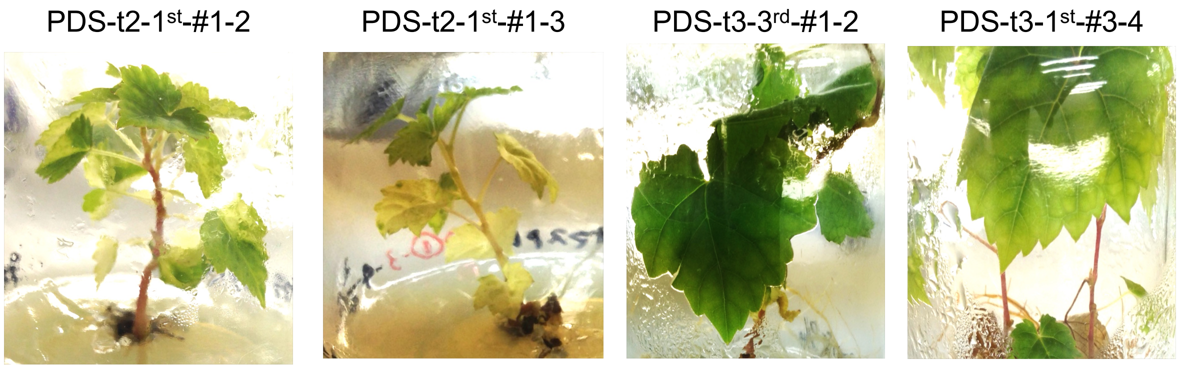

S4 Fig. Appearance of regenerated plants used for Western blot analysis.
Bleaching or pale green cells were dominant in three regenerated plans (PDS-t2-1st-#1-2, 3 and PDS-t3-1st-#3-4). On the other hand, leaf color of PDS-t3-3rd-#1-2 is almost the same as that of wild-type, 'Neo Muscat' (S2 Fig.).
